# Data of resistance to the compression of restorative dental materials

**DOI:** 10.1016/j.dib.2019.103755

**Published:** 2019-03-06

**Authors:** Antonio Díaz-Caballero, Arnulfo Tarón-Dunoyer, Adel Martínez-Martínez

**Affiliations:** a Department of Oral Medicine, Gitouc Research Group, School of Dentistry, University of Cartagena, Cartagena, Colombia; b Department of Food Science, Gibae Research Group, School of Engineering, University of Cartagena, Cartagena, Colombia

## Abstract

This article contains data related to the research article titled “Compressive strength of glass ionomer and composite resin. In vitro study” (Blanco et al., 2017). The presented data gives information about the compressive strength of two restorative materials (Tetric N-Ceram^®^ resin and Vitremer TM^®^ resin) in class I cavities in birradicular premolars. The dental organs were subjected to a compressive vertical force at different deeps of cavities preparation until obtain fracture of the restorative material. Compression Resistance values of 380.5 are obtained; 387; 399.2; 419.9; 422.4; 428; 432.1 and 429 N for Tetric N-Ceram^®^ and 349.4 resin; 350.2; 356.1; 369; 390.7; 416.2; 424 and 426.8 for the Vitremer TM^®^ ionomer. There are significant statistical differences (p < 0.05) between restorative materials. The resin Tetric N-Ceram^®^, showed higher resistance to compression than Vitremer TM^®^ resin in all depths.

**Table:** Specifications table

Subject area	Dentistry, Materials Science.
More specific subject area	Resistance of materials
Type of data	Tables
How data was acquired	The compressive fracture force data of restorative materials were obtained using a texturometer EZ-S SHIMADZU, series number 346-54909-33, with 50–60 Hz, with maximum capacity range of 500 N.
Data format	Observations, analyzed.
Experimental factors	A comparative-descriptive analysis of the compressive resistance to vertical forces of the Tetric N-Ceram^®^ and VitremerTM^®^ dental use resins was carried out. The fractures of the restorative material were made at different depths in the dental organ cavity using texturometry equipment.
Experimental features	The published data are used to determine the hardness of the restorative material when it is subjected to a vertical compressive force at different depths in the cavity of the dental organ.
Data source location	Faculty of Dentistry. University of Cartagena. Cartagena. Colombia.
Data accessibility	Data are available in this article
Related research article	Blanco, L.S., Frias, T.S., Tarón, D.A., Bustillo, A.J., and Diaz, C.A Compressive strength of glass ionomer and composite resin. In vitro study. Revista Odontológica Mexicana. 21 (2) (2017): 107–111. https://doi.org/10.1016/j.rodmex.2017.05.015 https://www.sciencedirect.com/science/article/pii/S1870199X17300411

**Table:** 

**Value of data** • Data of resistance to vertical compressive forces may be used to determine the hardness of restorative materials for dental use. The experimentation model can be replicated for resistance studies of materials and instrumental used in the dental area. • Data on the hardness of the resins allow to make comparisons in terms of quality with other restorative materials currently used.

## 1 Data

In this investigation, the values of vertical compression stress resistance in Tetric N-Ceram^®^ and Vitremer TM^®^ restorative materials were determined experimentally at different depths in sealed dental cavities [1]. In Table 1, Table 2, the data on the compression efforts of the samples may be observed. The dental organs with 4 mm deep cavities restored with Tetric N-Ceram^®^ resin reported to have greater hardness than those restored with Vitremer TM^®^ (see Table 3, Table 4).

**Table 1:** Experimental data of the values of vertical compressive strength (hardness) of the restorative material Tetric N-Ceram^®^.

Vertical compressive strength (Newton)								
Experience	Depth of the cavity (mm)							
	0.5	1	1.5	2	2.5	3	3.5	4
1	380.1	387.0	400.0	420.0	422.3	428.3	432.0	439.1
2	380.0	387.0	401.0	421.0	422.1	428.1	432.1	439.1
3	380.1	387.1	400.1	419.9	422.3	428.0	432.1	438.9
4	380.5	387.0	399.1	419.9	422.3	428.0	432.1	439.0
5	380.3	387.4	399.2	419.9	422.4	428.0	432.2	439.1
6	380.5	387.1	399.2	420.0	422.4	428.1	432.1	439.0
7	380.5	387.0	399.2	419.8	422.4	428.3	432.1	439.0
8	380.4	387.1	399.1	419.9	422.1	428.3	432.2	439.0
9	380.5	387.1	399.2	419.8	422.4	428.0	432.6	439.1
10	380.9	387.3	399.1	420.0	422.0	428.0	432.1	439.0
11	380.1	387.0	399.1	420.1	422.4	428.0	432.0	439.2
12	380.5	387.0	399.2	419.7	422.3	428.3	432.2	439.1
13	380.5	387.2	399.2	419.9	422.5	428.1	432.1	439.0
14	380.5	387.1	399.0	420.0	422.4	428.0	432.1	439.0
15	380.4	387.0	399.2	419.8	422.4	428.3	432.3	439.2

**Table 2:** Experimental data of the values of vertical compressive strength (hardness) of the restorative material Vitremer TM^®^.

Vertical compressive strength (Newton)								
Experience	Depth of the cavity (mm)							
	0.5	1	1.5	2	2.5	3	3.5	4
1	349.4	350.3	356.0	368.9	391.0	416.2	423.8	426.6
2	349.2	350.2	356.4	369.0	390.5	416.2	423.9	425.9
3	349.0	350.3	356.0	369.7	390.9	416.1	423.9	425.8
4	349.4	350.3	356.1	368.8	390.9	416.4	423.8	426.8
5	349.4	350.2	356.1	368.9	390.8	416.2	423.6	426.8
6	349.4	350.2	356.1	369.1	390.7	416.2	423.9	426.7
7	349.5	350.2	356.2	369.0	390.7	416.3	424.0	426.9
8	349.4	350.1	356.0	369.3	390.7	416.1	424.0	426.8
9	349.2	350.3	356.0	367.9	390.7	416.1	424.0	426.8
10	349.2	350.2	356.1	369.0	390.7	416.2	423.9	426.9
11	349.1	350.2	356.1	369.1	390.9	416.2	424.0	426.8
12	349.4	350.1	356.1	369.1	390.7	416.2	424.0	426.7
13	349.4	350.1	356.1	369.0	390.9	416.2	424.0	426.8
14	349.2	350.3	356.0	369.1	390.7	416.1	424.9	426.8
15	349.3	350.2	356.1	368.9	390.8	416.2	424.0	426.7

**Table 3:** Statistical analysis data of the values of vertical compressive strength (hardness) of the restorative material Tetric N-Ceram®.

*Normal Statistical*								
*Depth of the cavity (mm)*								
	0.5	1	1.5	2	2.5	3	3.5	4
Number of values	14	14	14	14	14	14	14	14
Minimum	380	387	399	419.7	422	428	432	438.9
25% Percentile	380.3	387	399.1	419.8	422.3	428	432.1	439
Median	380.5	387.1	399.2	419.9	422.4	428.1	432.1	439
75% Percentile	380.5	387.1	399.2	420	422.4	428.3	432.2	439.1
Maximum	380.9	387.4	401	421	422.5	428.3	432.6	439.2
Mean	380.4	387.1	399.4	420	422.3	428.1	432.2	439.1
Std. Deviation	0.2269	0.124	0.5417	0.3118	0.146	0.1328	0.1447	0.08549
Std. Error	0.06064	0.03315	0.1448	0.08332	0.03902	0.03549	0.03867	0.02285
Lower 95% CI of mean	380.3	387	399	419.8	422.2	428	432.1	439
Upper 95% CI of mean	380.5	387.2	399.7	420.2	422.4	428.2	432.2	439.1
Sum	14	14	14	14	14	14	14	14
*Table Analyzed One-way analysis of variance*								
P value	<0.0001							
Are means signif. different?								
(P < 0.05)	Yes							
Number of groups	8							
F	101432							
R square	0.999							
Bartlett's statistic	70.44							
P value	<0.0001							
Do the variances differ signif. (P < 0.05)	Yes							
ANOVA Table	SS	df	MS					
Treatment (between columns)	46571	7	6653					
Residual (within columns)	6.821	104	0.06559					
Total	46578	111

**Table 4:** Statistical analysis data of the values of vertical compressive strength (hardness) of the restorative material Vitremer TM^®^.

Normal Statistical								
Depth of the cavity (mm)								
	0.5	1	1.5	2	2.5	3	3.5	4
Number of values	15	15	15	15	15	15	15	15
Minimum	349	350.1	356	367.9	390.5	416.1	423.6	425.8
25% Percentile	349.2	350.2	356	368.9	390.7	416.1	423.9	426.7
Median	349.4	350.2	356.1	369	390.7	416.2	424	426.8
75% Percentile	349.4	350.3	356.1	369.1	390.9	416.2	424	426.8
Maximum	349.5	350.3	356.4	369.7	391	416.4	424.9	426.9
Mean	349.3	350.2	356.1	369	390.8	416.2	424	426.7
Std. Deviation	0.141	0.0743	0.1033	0.3681	0.128	0.0798	0.2783	0.3357
Std. Error	0.036	0.0191	0.0266	0.0950	0.0330	0.0206	0.0718	0.0866
Lower 95% CI of mean	349	350.2	356	368.8	390.7	416.1	423.8	426.5
Upper 95% CI of mean	349.5	350.3	356.2	369.2	390.8	416.2	424.1	426.8
Sum	5239	5253	5341	5535	5862	6243	6360	6400
*Table Analyzed One-way analysis of variance*								
P value	<0.0001							
Are means signif. different? (P < 0.05)	Yes							
Number of groups	8							
F	349338							
R square	1							
Bartlett's statistic	71.95							
P value	<0.0001							
Do the variances differ signif. (P < 0.05)	Yes							
Brown-Forsythe test								
F (DFn, DFd)	1326 (7, 112)							
P value	0,2445							
P value summary	Ns							
Are SDs significantly different (P < 0.05)	No							
ANOVA Table	SS	df	MS					
Treatment (between columns)	117552	7	16793					
Residual (within columns)	5.384	112	0.04807					
Total	120558	119						

## 2 Experimental design. Materials and methods

### 2.1 Materials

Were used Tetric N-Ceram^®^ resin and Vitremer TM^®^ ionomer brands of dental use acquired from existing commercial houses in the market.

### 2.2 Experimentation

In vitro experimentation was carried out in which vertical compressive strength of a Vitremer TM®- tested contrasting glass reconstructive ionomer was evaluated against a nano-hybrid composite resin (Tetric N-Ceram^®^). Commonly used to restore class I cavities with depths 0.5–4 mm (in 0.5 mm intervals) in human bi-radicular premolars [2], [3]. All teeth of the sample were subjected to stress tests with the EZ-S SHIMADZU texturometer with serial number 346-54909-33 of 50–60 Hz with a maximum capacity range of 500 N. Sealed and restored teeth received underwent compression on the occlusal side. It is emphasized that the application of the force was performed in the central point of the restoration for all samples in the same way [1].

### 2.3 Statistical analysis

The significance of means within the groups of experimental data was evaluated using one-way analysis of variance (one-way ANOVA).
