# Supplementary material for: Data of resistance to the compression of restorative dental materials
Source: Data Brief. 2019 Mar 6;23:103755. doi: 10.1016/j.dib.2019.103755 (PMC6660634; doi:10.1016/j.dib.2019.103755)
Supplement: Supplementary file 1 — Multimedia component 1 [file mmc1.pdf]

Universidad
de Cartagena
Fundada en 1827

Acreditación institucional de Alta Calidad
Resolución 2583 del 26 de febrero de 2014. Ministerio de Educación Nacional

Editor of *DATA IN BRIEF*

5 of January 2019

Dear Editor:

Very respectfully we are sending of the manuscript entitled *"**Data of resistance to the compression of restorative dental materials**"*. Which we would like to be considered for publication in *DATA IN BRIEF*.

We wish to confirm that there are no known conflicts of interest associated with this publication and there has been no significant financial support for this work that could have influenced its outcome.

We confirm that the manuscript has been read and approved by all named authors and that there are no other persons who satisfied the criteria for authorship but are not listed. We further confirm that the order of authors listed in the manuscript has been approved by all of us.

We confirm that we have given due consideration to the protection of intellectual property associated with this work and that there are no impediments to publication, including the timing of publication, with respect to intellectual property. In so doing we confirm that we have followed the regulations of our institutions concerning intellectual property.

We understand that the Corresponding Author is the sole contact for the Editorial process (including Editorial Manager and direct communications with the office). He is responsible for communicating with the other authors about progress, submissions of revisions and final approval of proofs. We confirm that we have provided a current, correct email address which is accessible by the Corresponding Author and which has been configured to accept email from: adiazc1@unicartagena.edu.co

Signed by all authors as follows:

Prof. Antonio Díaz Caballero. Ph.D.

Prof. Arnulfo Tarón Dunoyer. MSc.

Prof. Adel Martinez Martinez . MSc.

ISO 9001

icontec internacional

SC-CER153470

Departamento de Postgrado - Facultad de Ingeniería
Piedra de Bolívar – Av. Del Consulado, Calle 30 No. 48-152
Edificio Bloque de Biblioteca 2do piso Of F-202 Teléfax: (572) 669 9173
E-mail: pcingenieria@unicartagena.edu.co **web: www.unicartagena.edu.co**
Cartagena de Indias, D.T y C – Colombia
